# A discrepancy of *Chlamydia trachomatis *incidence and prevalence trends in Finland 1983–2003

**DOI:** 10.1186/1471-2334-8-169

**Published:** 2008-12-18

**Authors:** Erika Lyytikäinen, Marjo Kaasila, Eija Hiltunen-Back, Matti Lehtinen, Kaisa Tasanen, Heljä-Marja Surcel, Pentti Koskela, Jorma Paavonen

**Affiliations:** 1 National Public Health Institute, Oulu and Helsinki, Finland; 2 Deparment of Dermatology, University of Oulu and Oulu University Hospital, Oulu, Finland; 3 Department of Dermatology and Venereology, Helsinki University Hospital, Helsinki, Finland; 4 University of Tampere, School of Public Health, Tampere, Finland; 5 Department of Obstetrics and Gynecology, University of Helsinki, Helsinki, Finland

## Abstract

**Background:**

Reported rates of *Chlamydia trachomatis *are on the rise contradicting the declining rates of *C. trachomatis *associated reproductive sequelae in Western countries. Population based evaluation of the real trend of *C. trachomatis *infection is important to contemplate prevention efforts. We studied *C. trachomatis *occurrence during the past 20 years in Finland comparing incidence rate data based on serology and reported *C. trachomatis *laboratory notifications.

**Methods:**

A random sample of 7999 women with two consecutive pregnancies within five years was selected from the population of the Finnish Maternity Cohort (FMC) serum bank stratified by calendar year and age. *C. trachomatis *IgG antibodies were determined by a standard peptide-ELISA. The reported incidence rates of *C. trachomatis *infections based on case notifications were obtained from the National Registry of Infectious Diseases (NIDR).

**Results:**

*C. trachomatis *seroprevalence rates decreased significantly from 1983 to 2003 both in women under 23 years of age (23.3% to 9.2%) and in women between 23–28-years of age (22.2% to 12.6%). However, seroconversion rates increased from 31 per 10000 person years in 1983–85 to 97 per 10000 person years in 2001–2003 (incidence rate ratio 3.2, 95% CI, 1.1–8.7) among the older age group. Seroconversion rate was highest (264) in 1983–1985 in the younger age-group, then declined and subsequently increased again (188) in 2001–2003. The incidence based on seroconversions was in agreement with the reported incidence rates in both age groups.

**Conclusion:**

*C. trachomatis *seroprevalence rate decreased during 1983–2003 among fertile-aged women in Finland. During the same time period incidence rates based both on seroconversions and reported laboratory notifications of diagnosed *C. trachomatis *infections increased. The discrepancy between the *C. trachomatis *incidence and seroprevalence trends warrants further studies.

## Background

*Chlamydia trachomatis *is a major threat to the reproductive health of women [1]. National surveillance programs have largely failed to reduce the disease burden caused by *C. trachomatis *[2]. In the Nordic countries, incidence rates have increased between 1999 and 2005 [3-5]. In Finland, the incidence of reported *C. trachomatis *infections increased by 60% over the last 10 years, peaking in the age group of 15–24 [6]. Since most chlamydial infections are asymptomatic [7], true incidence may well be higher than that based on reports.

In a population based study, we recently reported declining *C. trachomatis *antibody prevalence rates [8]. The decrease was countrywide although small clusters of high prevalences were seen around large cities and in the Southeastern part of the country [8]. Similar trend has been reported from Japan [9]. These reports suggest that the control efforts may have been efficient.

To clarify the discordance between the reported infection rates to the National Infectious Disease Register (NIDR) and the seroprevalence rates, we studied the incidence rates of *C. trachomatis *infection by serology using paired serum bank samples. In order to have a better understanding of the real trend of *C. trachomatis *infections in the population, we compared incidence rate data based on serology and reported *C. trachomatis *laboratory notifications.

## Methods

### Finnish Maternity Cohort

More than 98% of pregnant women in Finland (altogether 750000) have participated in the serological screening for congenital infections (syphilis, HIV, and hepatitis B) during the first trimester. Approximately 50% of the women become pregnant again within 5 years of the first pregnancies and donate 2^nd ^blood samples to the Finnish Maternity Cohort (FMC) serum bank. The blood samples have been stored in FMC serum bank at the National Public Health Institute (KTL) since 1983 and the FMC serum bank comprises approximately 1.5 million serum samples.

A total of 275 505 women (< 29 years of age) with paired serum samples were identified for *C. trachomatis *antibody testing as previously described [8]. The FMC cohort was divided into 28 strata according to calendar year (by 3 year periods i.e. 1983–1985, 1986–1988, 1989–1991, 1992–1994, 1995–1997, 1998–2000, 2001–2003) and age (< 20, 20–22, 23–25 and 26–28) as described [8]. A random subsample of 200 or 400 women in each stratum was obtained. A total of 401 women were excluded because of missing data. Eventually, 7999 women were tested for *C. trachomatis *IgG-antibodies (Table 1).

**Table 1:** Number of women (n) with at least 2 pregnancies within 5 years selected randomly by age and calendar time of possible seroconversion

**Age**	**1983–85** **n**	**1986–88** **n**	**1989–91** **n**	**1992–94** **n**	**1995–97** **n**	**1998–00** **n**	**2001–03** **n**	**Total**
< 20	194	190	198	193	177	184	184	1320
20–22	195	195	193	199	187	194	185	1348
23–25	390	390	388	394	376	365	362	2665
26–28	393	397	384	389	366	367	370	2666
Total	1172	1172	1163	1175	1106	1110	1101	7999

The study was approved by the institutional ethical committee and the FMC steering committee.

### National Infectious Disease Register

Surveillance of sexually transmitted *C. trachomatis *infections is based on mandatory notifications according to a Communicable Diseases Act and Decree of 1987. Between 1987 and 1997, confirmed *C. trachomatis *positive cases were reported to the NIDR by physicians, but from 1997, by laboratories only (between 1995 and 1997 by both). The laboratory notification includes personal identification number (PIN), gender, age, place of sampling and test used. Data are updated weekly. Same person may have been reported more than once if the time interval exceeded three months. Data after 1995 can be accessed at  and is available in fixed subgroups of age, time period of diagnosis and gender. Data from earlier years 1987–1994 have been reported elsewhere [10].

*C. trachomatis *incidence rates were recorded in two age groups (≤ 24, and 25–29-year-olds) in four different time periods (1995–1997, 1998–2000, 2001–2003, 2004–2006).

### Serology

Serum IgG antibodies to *C. trachomatis *were analyzed in a single set of experiments by commercial enzyme immunoassay (EIA), as previously described [8]. The results were expressed as mean absorbance (optical density 450 mm) of duplicated samples minus the mean absorbance of the reagent blank divided by the cut-off value. A value of greater than 1.4 was considered positive [8].

### Statistical analysis

Two youngest and two oldest age groups were combined for statistical analyses into two categories (women below 23 years and those between 23 and 28 years of age; Table 1). Baseline samples (first pregnancy serum sample) were used to calculate *C. trachomatis *seroprevalence rates. Differences in seroprevalence rates were tested using binomial test for differences in proportions. All 6632 women who were seronegative at the baseline were eligible for analysing the incidence rates. *C. trachomatis *incidence data was based on seroconversions i.e. the second sample of the baseline seronegative woman was positive. Incidence rates were estimated by the number of seroconversions divided by person years of follow-up. The time for each seroconversion was assumed to be in the midpoint of the two samplings. Crude incidence rate ratio with 95% confidence interval was calculated. Statistical analyses were performed using R 2.6.0 (R Development Core Team, Vienna, Austria) and SPSS 15.0 (SPSS Inc., Chicago, Illinois, USA).

## Results

### *C. trachomatis *seroprevalence trends

**Figure 1:**
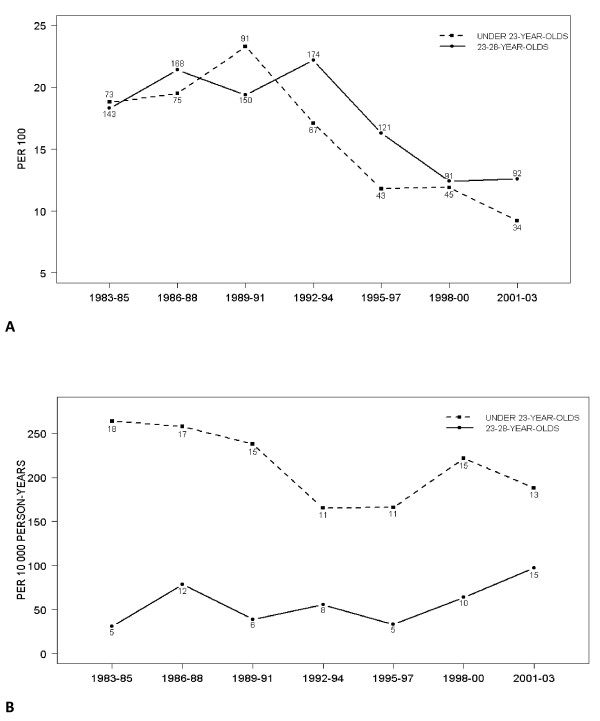
***Chlamydia trachomatis *****a) seroprevalence (%) with the number of prevalent cases and b) incidence (per 10 000 person years) with the number of seroconversions in pregnant women during 1983–2003.**

*C. trachomatis *seroprevalences did not differ between women who were < 23 years of age and those between 23 and 28 years of age (Figure 1A) as their 95% confidence intervals overlapped at each calendar time point (data not shown). The highest *C. trachomatis *seroprevalence rates of 23.3% (CI 19.1–27.5) and 22.2% (19.3–25.1) occurred in 1989–1991 in the younger age group and in 1992–1994 in the older age group. Thereafter, the rates declined significantly (p < 0.001) being 9.2% (6.3–12.2) in 2001–2003 in the younger group and 12.4% (10.0–14.8) in 1998–2000 in the older age group.

### *C. trachomatis *incidence rates based on seroconversions

A total of 161 seroconversions occurred among 6632 (2.4%) women who were seronegative at baseline. The rate of seroconversions varied from 3.4% to 5.7% in the younger age group and from 0.8% to 2.3% in the older age group.*C. trachomatis *incidence rates were higher in women who were < 23 years of age than in women between 23 and 28 years of age through out the whole time period although the difference decreased towards 2003 (Figure 1B).

*C. trachomatis *incidence rate based on seroconversions was 214 (95% CI 172–256) per 10000 person years in the younger age group and 57 (CI 43–71) per 10000 person years in the older age group. In the younger age group the incidence was highest (264 per 10000 person years, CI 142–386) at the beginning of the 1980s (Figure 1B) and lowest (166 per 10000 person years, CI 68–264) between 1992 and 1994. In 2001–03 the incidence rate increased to 188 (CI 86–290). However, no statistically significant time trends were observed.

In the older age group, the incidence was lowest (31, CI 3.8–58) between 1983 and1985. The incidence increased to 97 (CI 48–146) in 2001–2003 (not significant linear trend, p = 0.10). The relative difference in the incidence rates between 1983–1985 and 2000–2003 was significant (rate ratio 3.2, 95% CI, 1.1–8.7).

### *C. trachomatis *incidence rates based on laboratory notifications

The point estimates based on the rates of the NIDR notifications were in line with *C. trachomatis *incidence rates based on seroconversions (Table 2). According to the laboratory notifications, *C. trachomatis *incidence increased in both age-groups (< 24 and 25–29 years of age) since 1995–1997 (Table 2). The incidence rates were highest in women below 24 years of age with 1.5-fold increase from 245 (95% CI 237–253) between 1995 and 1997 to 374 (95% CI 365–383) between 2004 and 2006. A moderate 1.3-fold (CI 1.21–1.43) increase from 57 (CI 53–61) to 70 (CI 66–75) was noted in the women between 25 and 29 of age.

**Table 2:** *Chlamydia trachomatis *incidence rates per 10 000 person years (95% confidence interval) based on serology or laboratory reports

		**Time period**		
	***Age group***	***1995–1997***	***1998–2000***	***2001–2003***

FMC	< 23-year-olds	166 (68 – 264)	222 (110 – 334)	188 (86 – 290)
	23–28-year-olds	33 (4 – 62)	64 (24 – 103)	97 (48 – 146)
				
NIDR	15–24-year-olds	245 (237–253)	307 (298–315)	374 (365–383)
	25–29-year-olds	57 (53 – 61)	64 (60 – 68)	70 (66 – 75)

FMC = Finnish Maternity Cohort; NIDR = National Infectious Disease Register

## Discussion

We showed that the *C. trachomatis *incidence rates based on serology were in line with the incidence rates based on laboratory notifications. The increasing incidence trends were at variance with decreasing *C. trachomatis *seroprevalence rates. Our results suggest that the true incidence rates can best be demonstrated by seroconversions detected in paired serum samples. The serum bank material used is likely to reveal the true population based incidence.

The number of cases with seroconversions followed the common u-shaped curve of reported *C. trachomatis *cases especially among the younger age group [1,4,10]. The conformity between the FMC and NIDR incidence rates indicates that the increasing trend is real. Testing of more individuals and using more sensitive diagnostic tests would not fully explain the increasing *Chlamydia *incidence rates.

The discrepancy between reported cases and seroprevalence rates is not easy to explain. Such discrepancy have earlier been reported from British Columbia during an extensive *C. trachomatis *control program [1]. Earlier diagnosis and treatment may lead to impaired immune response [1] which then reduces the overall seroprevalence rate in the population. Reinfection rates have not been analysed from the Finnish NIDR. However, increasing seroconversion rate suggests that the reported chlamydia rates are not explained merely by high reinfection rate.

The observed discrepancy between the incidence rate and seroprevalence is comparable with the declining trend of the long-term sequelae of *C. trachomatis *infection, e.g. PID, preterm delivery and ectopic pregnancy in Finland over the last ten years [11,12] and suggest that the infection burden in the population would be decreasing. However, it can not be excluded that the increasing incidence rate reflects changes in the serotype distribution or type replacement with less pathogenic strains over time. More research is needed to know whether this could be a consequence of improved diagnosis and efficient treatment of symptomatic infections caused by virulent *C. trachomatis *serotypes linked with stronger symptoms [13-17].

On the other hand, direct comparison of the FMC data and NIDR data is not straightforward. Most *C. trachomatis *infections occur in adolescents [5,18,19] with risk taking behaviour [20]. Although the FMC serum bank covers almost all pregnant women and is population based, it lacks infertile women and to a large part also adolescent women. Furthermore, contact tracing efforts probably lead to increasing test numbers and more asymptomatic cases reported to the NIDR.

Knowledge of *C. trachomatis *seroprevalence and incidence trends is extremely important since these trends reflect prevention efforts in the population. Furthermore, epidemiologic data are important not only for monitoring *C. trachomatis *infection trends but also for identification of breakthrough mutants [21]. A recent survey among elementary school students showed that the age of sexual debut is decreasing and condom use is inconsistent [22]. Increasing rates of reported *C. trachomatis *infection and the high incidence based on seroconversions fit into the picture. There is no national screening program in Finland although some maternity clinics routinely test women. Nevertheless, the Ministry of Social Affairs and Health has prepared a recommendation for opportunistic screening in Finland but significant reduction in the *C. trachomatis *rates is unlikely to take place unless systematic screening is implemented.

## Conclusion

This study confirms the decline of *C. trachomatis *seroprevalence in Finland since the middle of 1990s'. Simultaneously demonstrated rising seroconversion rates in different individuals are in accordance with the reported laboratory notifications in the NIDR suggesting that the increasing infection rates are not only due to repeated infections. Based on our results, population-based *C. trachomatis *incidence rates can be demonstrated by seroconversions detected in paired serum samples. The discrepancy between the *C. trachomatis *incidence and seroprevalence trends warrants further studies.

## Competing interests

The authors declare that they have no competing interests.

## Authors' contributions

EL made substantial contribution to the study design, interpretation of findings and writing of the manuscript; MK conducted data analysis, provided statistical advice and participated in writing of the manuscript; EH-B, ML and KT all participated in critical interpretation of findings and writing of the manuscript; HMS participated in overseeing laboratory issues, monitoring laboratory procedures, critical interpretation of findings and writing of the manuscript; PK conceived of the study and contributed to critical revision of the manuscript; JP participated in interpretation of the study results and gave final approval of the manuscript to be published.

## Pre-publication history

The pre-publication history for this paper can be accessed here:


